# Impact of Gamma Radiation on Dynamic R_DSON_ Characteristics in AlGaN/GaN Power HEMTs

**DOI:** 10.3390/ma12172760

**Published:** 2019-08-28

**Authors:** Pedro J. Martínez, Enrique Maset, Pedro Martín-Holgado, Yolanda Morilla, David Gilabert, Esteban Sanchis-Kilders

**Affiliations:** 1 Department Electronic Engineering, University of Valencia, 46100 Burjassot, Spain; 2 Centro Nacional de Aceleradores (CNA), Universidad de Sevilla, CSIC, JA, 41092 Sevilla, Spain

**Keywords:** high-electron-mobility transistor (HEMT), gallium nitride (GaN), radiation hardness, assurance testing, radiation effects, total ionizing dose (TID)

## Abstract

GaN high-electron-mobility transistors (HEMTs) are promising next-generation devices in the power electronics field which can coexist with silicon semiconductors, mainly in some radiation-intensive environments, such as power space converters, where high frequencies and voltages are also needed. Its wide band gap (WBG), large breakdown electric field, and thermal stability improve actual silicon performances. However, at the moment, GaN HEMT technology suffers from some reliability issues, one of the more relevant of which is the dynamic on-state resistance (R_ON_dyn_) regarding power switching converter applications. In this study, we focused on the drain-to-source on-resistance (R_DSON_) characteristics under ^60^Co gamma radiation of two different commercial power GaN HEMT structures. Different bias conditions were applied to both structures during irradiation and some static measurements, such as threshold voltage and leakage currents, were performed. Additionally, dynamic resistance was measured to obtain practical information about device trapping under radiation during switching mode, and how trapping in the device is affected by gamma radiation. The experimental results showed a high dependence on the HEMT structure and the bias condition applied during irradiation. Specifically, a free current collapse structure showed great stability until 3.7 Mrad(Si), unlike the other structure tested, which showed high degradation of the parameters measured. The changes were demonstrated to be due to trapping effects generated or enhanced by gamma radiation. These new results obtained about R_ON_dyn_ will help elucidate trap behaviors in switching transistors.

## 1. Introduction

Gallium nitride (GaN) is a promising material for next-generation power devices due to its wide band gap, which allows a large breakdown electric field and the possibility of operating under harsh environmental conditions [1,2,3]. Such characteristics make these devices promising for space applications, where temperature and radiation are key factors. Particularly, the development of new GaN high-electron-mobility transistors (HEMTs) with great characteristics, such as low on-resistance and parasitic capacitances, allow them to switch at higher frequencies with high efficiency, making them attractive. The inherent radiation hardness, the capability to withstand higher breakdown voltages, and the higher operating temperatures will enable this technology’s use in future space applications, such as telecommunications, Earth observation, and science missions [4,5].

These promising advantages have pushed the research focus on the reliability of GaN devices under radiation conditions. In space environments, energetic particles which impact semiconductor devices lose their energy to ionizing and nonionizing processes while they travel through the devices. The energy loss causes the production of electron–hole pairs (ionization) and displaced atoms (displacement damage). Gamma irradiation is one of the tests used to evaluate the hardness of devices to be used in aerospace applications. Our main objective here was to study the degradation induced by the total ionizing dose (TID) effects of ^60^Co γ-ray radiation on GaN HEMTs. The response to gamma irradiation is complex. Compton electrons induced by γ-radiation create electron–hole pairs, thus changing the occupancy of traps. Regarding this topic, many research papers have been published in recent years showing different behaviors depending on the dose applied and the structure of the HEMT being irradiated [6]. In general, HEMTs irradiated with gamma rays exhibit a negative threshold voltage and a transconductance decrease, which can be explained by the creation of trap states throughout the structure and, in some cases, an increase in the two-dimensional electron gas (2DEG) sheet concentration [7,8]. Some authors have reported strain relaxation at low-dose gamma irradiation, which enhanced the channel mobility [9,10]. In contrast, other authors [11] have reported a reduction of 60% of the drain current at around 70 krad(Si). Thus, the defects generated by the γ-irradiation are very sensitive to the structure, having defect creation rates dependent on the quality of the sample and the doping level.

On the other hand, it is well known that another problem related to the GaN HEMT structure is the electron trapping effects which decrease device performance [12], in particular, the dynamic on-resistance (R_ON_dyn_) [13]. This trapping reduces the current that the devices can drive below the device’s rated current and could be attributed to a trapping effect in different regions inside the device. In [14], it was reported that trapping could be attributed to the device surface and buffer layer and that it was possible to distinguish between both. These trapping effects have been shown to be induced by the electrical field applied between the drain and the gate. Additionally, it was confirmed that the HEMT structure design is a key factor regarding the R_ON_dyn_. Different strategies can be used to mitigate the increase of R_ON_dyn_, mainly, the use of a p-GaN region close to the drain that is electrically connected to the drain edge [15] and the optimization of the device buffer layer design [16].

Taking into account the problems regarding the effects of γ-radiation and R_ON_dyn_, it is necessary to evaluate the effect of γ-radiation on R_ON_dyn_ if GaN HEMTs are to be seriously considered for future space applications. In fact, international space agencies, such as the National Aeronautics and Space Administration (NASA) and the European Space Agency (ESA), have shown increasing interest in evaluating the development of GaN devices. Some testing activities have been included as part of a future radiation qualification process for wide band gap devices, as presented in [17].

Analyzing the relevant literature shows that some contradictory results have been reported and the reasons for these differences are not evident. Furthermore, a previous study reported changes in R_ON_dyn_ [18] with low-dose gamma radiation. Therefore, in this study, we investigated not only the static characteristics but also the R_ON_dyn_ behavior of two different HEMT structures subjected to gamma radiation until 3.7 Mrad(Si). New results on this subject are shown in this work related to the HEMT structure and the biasing conditions applied during irradiation. These results show that, depending on the structure and the bias applied, trapping can be increased (case of negative gate bias) or reduced (case of shorted devices), which provides more information than previous studies, where only static characterization had been done.

## 2. Materials and Methods 

Normally-off commercial AlGaN/GaN HEMTs on Si with a voltage rating of 600 V were used for the radiation experiment. We chose two different HEMT structures: One was a 600 V p-doped GaN gate injection transistor (HD-GIT) PGA26E07BA, manufactured by Panasonic, Inc. (Kadoma-shi, Osaka, Japan). The HD-GIT has an additional p-GaN region between the gate and the drain, in which the holes are injected during the off-state, compensating the electron trapping and avoiding R_ON_dyn_. The other was the GS66516T, a 650 V p-GaN gate with a metal insulator layer (MISHEMT), manufactured by GaN Systems, Inc. (Ottawa, ON, Canada). Table 1 summarizes the key parameters of the investigated devices. 

For the TID radiation experiment, 12 devices from each HEMT structure were used. As shown in Table 2, different bias conditions were applied during irradiation to evaluate the bias dependence of the TID response of the HEMTs. In addition, one sample of each device type was selected as a reference (control device) to confirm the proper operation of the measurement system; that is, these unirradiated devices were subjected only to electrical measurements after each step, without any bias or radiation conditions applied.

The test campaign was carried out in the CNA-RadLab facility at the National Center for Accelerators in Seville, Spain. The gamma irradiation contained a ^60^Co gamma source with associated photon energies of 1.17 and 1.33 MeV (mean value: 1.25 MeV). The selected dose rate was 23.742 krad(Si)/h, which is within the “standard rate” window (0.36–180 krad(Si)/h) of the ESA, according to the TID Test Method [19]. The dose rate was obtained by measuring the charge with two TM30013 ionization chambers (PTW-FREIBURG, Germany) and one multichannel electrometer, MultiDOS (PTW-FREIBURG, Germany), and also considering the environmental correction factor. The dose rate uniformity in the filter box was 98.5%. The devices under test (DUTs) were mounted on a printed circuit board which was placed into a 12 × 17 cm filter box to be subjected to radiation, in compliance with the European Space Components Coordination Basic Specification No. 22900 [19]. This container had 2 mm of aluminum and 1.5 mm of lead in the outer layer and a 5 mm front cover of polymethyl methacrylate (PMMA) to achieve the charged-particle equilibrium.

Six irradiation steps were carried out during the campaign. Post-irradiation electrical measurements were performed after each exposure step for all the devices, including the control devices. At the end of total irradiation, two annealing steps were implemented. The first step consisted of room-temperature annealing under bias for 24 h. Afterwards, accelerated aging was carried out, where the devices were baked at 100 ± 5 °C under bias for 168 h. In both annealing steps the bias voltage applied on each DUT was the same as that during the irradiation steps.

**Figure 1:**
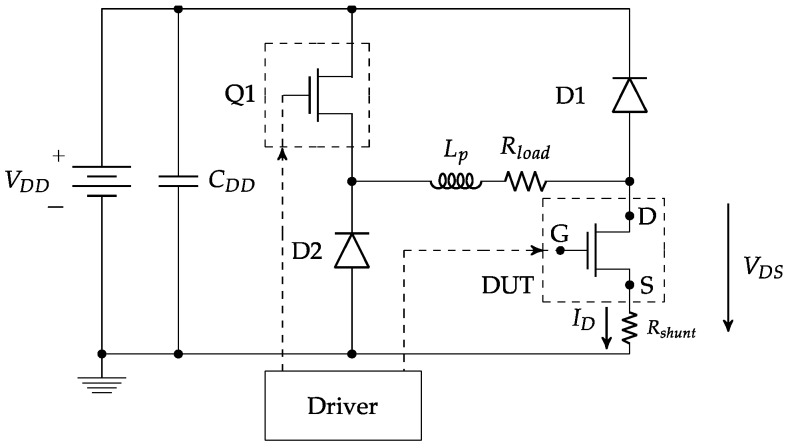
Circuit diagram used to measure R_ON_dyn_.

Concerning the measurements, two types were performed: I–V measurements were done with a Keysight Power Device Analyzer B1505A (Santa Rosa, CA, USA), and for the R_ON_dyn_ measurements, a custom circuit (Figure 1) was implemented [20].

The implemented switching circuit had the benefit of fully controlling the time that the voltage stress was applied to the GaN HEMT. Basically, it consisted of two transistors connected in series between the drain and the source, with a resistive load between both of them. Transistor Q1 was used to control the stress/trapping time. A resistive load R_load_ was used to set the current level when DUT was in on-state. Due to the inherent parasitic inductance (L_p_) of the power R_load_, two SiC diodes (C4D05120)—D1 and D2—offered a freewheeling path for the current when either Q1 or DUT was switched from on to off.

For the transistor Q1, a SiC MOSFET (C3M0065090) (Wolfspeed, Durham, NC, USA) was used in order to have a low output capacitance (C_DS_) and low current peaks due the charge and discharge of this parasitic capacitance. The values of the drain-to-source on-resistance (R_DSON_) of the DUT were obtained by measuring the device on-state voltage (V_DSON_) across it and dividing by the current (I_DS_) through the DUT. The drain current was measured using a coaxial shunt resistor of 98 mΩ (SDN-414-10), and for the V_DS_, a 300 V and 500 MHz passive voltage probe was selected.

Due to the high voltage applied to the DUT, the voltage across it represents a large dynamic range input signal for the oscilloscope input amplifier acquisitions, which can be overloaded, and as a result, an accurate determination of the on-state voltage would not be achievable. To avoid that problem, a voltage clamp circuit together with the passive voltage probe was used. In particular, the voltage clamp used was the commercial clp1500V15A1 from Springburo GmbH Emmendingen, Baden-Wurtemberg, Germany). The low range (2 V) was selected in the voltage clipper in order to have a faster response—200 ns in this case—considering the passive voltage probe and the voltage clipper. Precise frequency response compensation was done in the passive voltage probe to make up for the whole chain of the clipper and the voltage probe.

To control the “on-time” of the DUT, a generic MOSFET isolated driver SI8271BB (Silicon Labs, Austin, TX, USA) was used. This driver was selected due to the minimum supply voltage needed of 3 V. This low gate voltage was required to drive GaN devices with V_G_ = 4 V, which allowed us to see any change in the trapping charges when measuring the R_ON_dyn_. This is because, at a higher gate voltage, the 2DEG density at the AlGaN/GaN interface is higher, so the device is able to drive low drain currents without being affected by the trapped charges in the surface or the buffer. Otherwise, if we had used a lower gate voltage value, the density of the 2DEG would decrease and we could see any changes in the R_ON_dyn_ due to the trapping, even at low currents. Thus, in both cases, the trapped charges are present; however, in the case of the higher gate voltage, it would require higher currents to see the effect by measuring the R_ON_dyn_. Thus, instead of increasing the current, which could induce other problems such as self-heating, which would change the dynamic response, we chose to use a lower gate voltage to see any changes in the device trapping.

## 3. Experimental Results

The effect of gamma radiation on both tested structures was different. In this section, we analyze the changes of the static and dynamic characteristics considering the effects of applied bias conditions due to the potential increase of the concentration of activated GaN defects [21].

### 3.1. Transfer Characteristics

**Figure 2:**
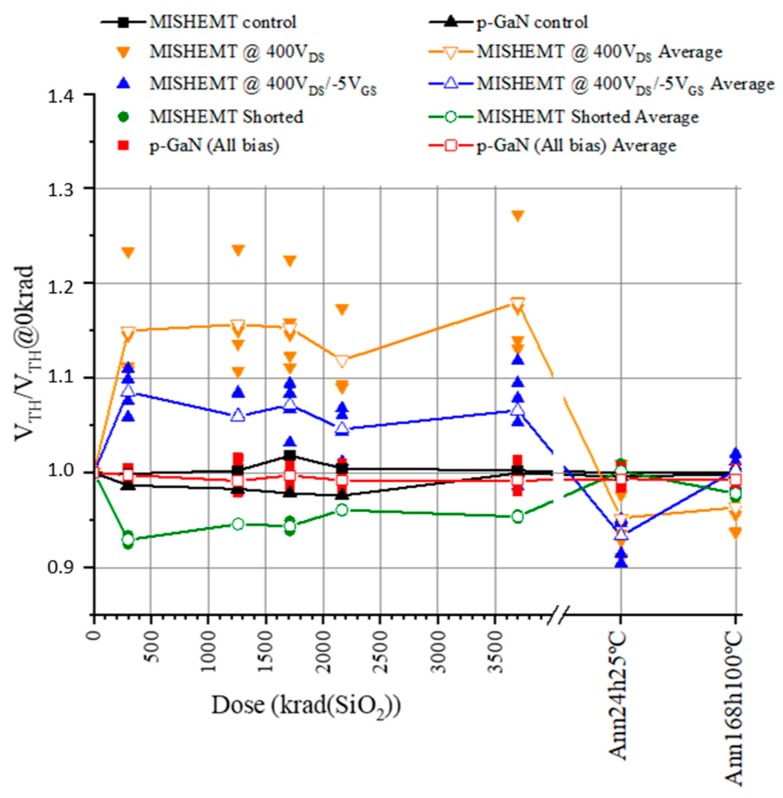
Normalized threshold voltage of GaN HEMTs as a function of total dose.

The main electrical measurements address the drain and gate currents, which are expressed as a function of the gate and drain voltages. From the I_DS_–V_GS_ characteristics, different parameters can be established to provide information about the changes suffered by the GaN HEMTs depending on the radiation applied. The first parameter analyzed was the threshold voltage (V_TH_), which was extracted by the current extrapolation method, where the threshold voltage value is the V_GS_ value corresponding to I_DS_ = 100 mA with a V_DS_ = 0.1 V. The threshold voltage is usually measured at lower currents; however, our setup and the limits defined for the test (until 3 A) forced us to use a higher current value (100 mA) to have better precision for threshold voltage measurement. Figure 2 shows the variations of the threshold voltage for all the devices. It is important to highlight the way the results are plotted, since each device had a different V_TH_ value. To provide a comparison, it was necessary to normalize this value, dividing the V_TH_ of each device at each step between the corresponding initial V_TH_, measured at 0 krad(Si).

The HD-GIT HEMT devices did not suffer any change with the accumulated dose, but the MISHEMT devices displayed different behaviors depending on the bias applied during the irradiation process. The devices subjected to drain and gate voltage during irradiation presented a positive increment of V_TH_ between 5% and 10%, while the devices stressed only with a positive voltage at the drain experienced a higher positive drift of V_TH_ between 10% and 25%. In contrast, the devices subjected to radiation with shorted pins suffered a negative variation of V_TH_ around 5–8%. In all cases, once the high-temperature annealing step of 168 h was finished, all the devices almost fully recovered their original values. 

This negative shift of V_TH_ in devices shorted during irradiation and the positive shift of the devices with bias applied matched with the findings reported in [8,22]. The negative shift was due to the trapped holes in the gate dielectric or the interface with AlGaN. The recovery of V_TH_ during the high-temperature annealing is explained by the release of trapped holes in the AlGaN layer and/or their neutralization by electrons from the channel. Higher temperatures resulted in higher hole mobility, allowing the holes to migrate or recombine and, therefore, reducing the hole impact on device operation. Meanwhile, during irradiation with positive bias applied, both hole and electron trapping took place. However, due to the high voltage applied, the electrons filled the hole traps, and in this way, the electron traps dominated in assisting the positive shift of the devices subjected to bias voltage during irradiation. 

**Figure 3:**
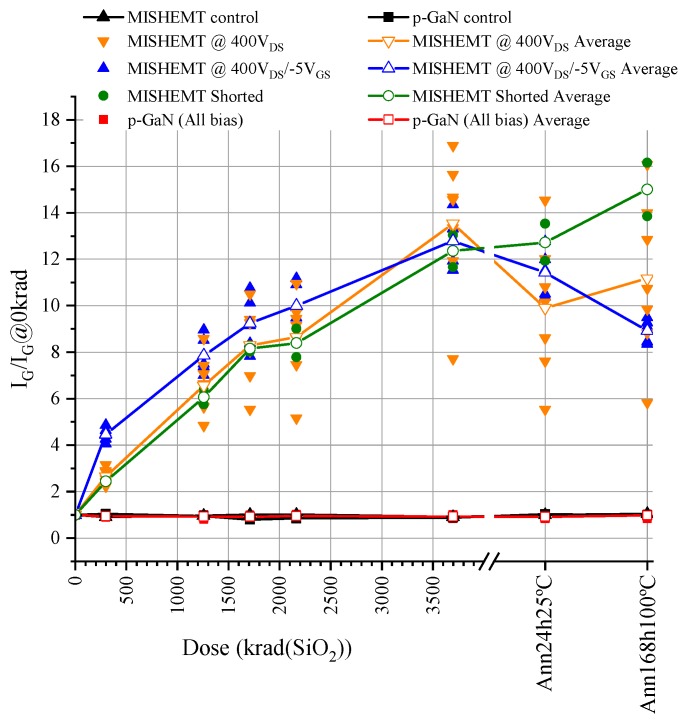
Normalized forward gate current (I_GS_) of GaN HEMTs as a function of the total dose measured at V_GS_ = 3V.

More information can be extracted with the second parameter analyzed, which is the variation over the gate characteristics I_GS_–V_GS_ (input characteristic), which is a way to evaluate the quality of the gate contact. Figure 3 shows the evolution of I_GS_ over the accumulated dose for all the devices measured at the maximum gate voltage applied of 3 V. 

As shown in Figure 3, the HD-GIT devices did not suffer any changes, while the GaN MISHEMT devices suffered a high increase in the I_GS_ without dependency on the bias applied during irradiation. This increase was proportional to the accumulated dose and independent of the bias condition applied. This forward gate current did not recover its initial value after the annealing process. Only a slight recovery around 10% was observed for the stressed devices, while shorted MISHEMT devices still degraded after the high-temperature annealing.

Increases in I_GS_ can be explained by radiation-induced defects on the gate insulator. The gate insulator in MISHEMT is used to make normally off devices, which depletes the channel while greatly reducing the gate current. However, the defects created during irradiation reduce the effectiveness of this insulator, and holes can become trapped in these defects, thus increasing the gate current. This also explains the negative shift of the threshold voltage mentioned above in the shorted devices. 

**Figure 4:**
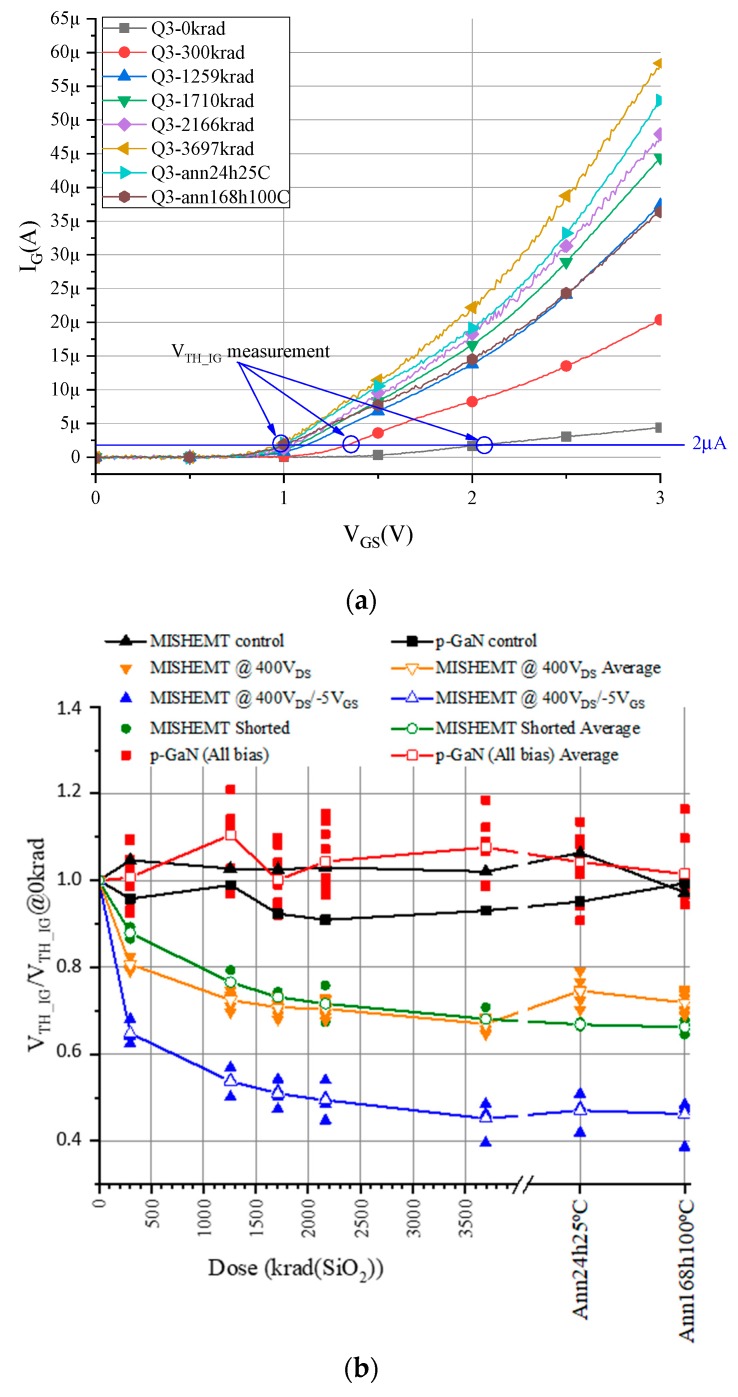
(**a**) Gate Schottky diode characteristics I_GS_–V_GS_ (input characteristic) for V_DS_ = 0.1 V. (**b**) Normalized threshold voltage measured for a gate current of 2 μA.

Focusing on MISHEMT devices and measuring the increase of I_GS_ has shown that there are no differences due to bias applied, but one difference was detected at this work, which is the displacement of I_G_–V_GS_ characteristics with the accumulated dose. In order to measure this displacement, the gate voltage was measured when the gate current crossed 2 mA, which we refer to as V_TH_IG_ to differentiate it from the V_TH_. In Figure 4a, blue markers show how this voltage was measured, and the normalized values are shown in Figure 4b for all the devices. This figure demonstrates that there is a clear difference between MISHEMT devices subjected to irradiation with negative gate voltage and the other MISHEMTs. There was a significantly larger negative shift in the V_TH_IG_ in the devices subjected to radiation with a negative gate bias. This difference can be explained by the additional electron traps created during irradiation in the region under the gate due to the reduced barrier heights of the traps when negative bias was applied at the gate. This induced an increase in the gate current for the low gate voltages due to the need for gate current injection to remove the traps.

### 3.2. Off-State Drain Current Measurement

**Figure 5:**
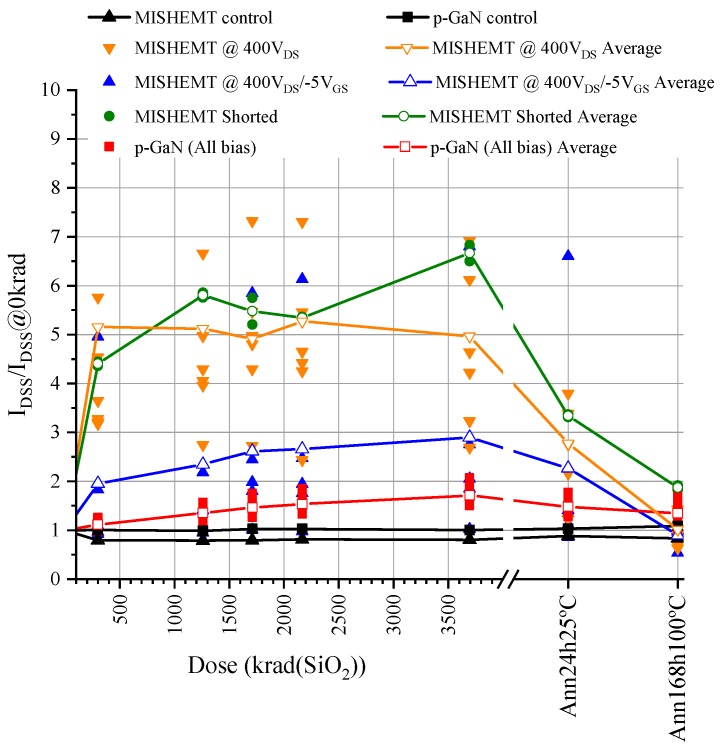
Normalized drain-to-source leakage current when applying a positive gate voltage of 500 V at the drain with 0 V at the gate.

In each step during the radiation test, the drain-to-source leakage current was also measured. The value of the leakage current measured at 500 V of drain while the gate was biased with 0 V is shown in Figure 5. The HD-GIT HEMT irradiated samples suffered a small increase in the drain leakage current; conversely, the GaN MISHEMT devices experienced a much larger increase, which was seven times higher than its value before irradiation. The leakage currents between the drain and the source increased with the accumulated dose during irradiation. Furthermore, they recovered approximately their initial values after the high-temperature annealing process. This increase in leakage current matched the gate insulator degradation, which favored hole trapping due to the reduced barrier height of the traps, and it reduced the effectiveness of the channel depletion. Additionally, this increase in drain leakage current was lower in the devices subjected to negative gate voltage during irradiation. This was due to the compensation of the hole traps by the existence of additional electron traps under the gate induced by the negative gate bias during radiation.

### 3.3. Dynamic Resistance Measurement

In order to evaluate the R_ON_dyn_ of the devices subjected to radiation, two different tests were performed: the double-pulse test (DPT) and the multi-pulse test (MPT). Both tests were conducted with the circuit setup shown in Figure 1, and the driving signal test sequence for each test is detailed in Appendix A.

**Figure 6:**
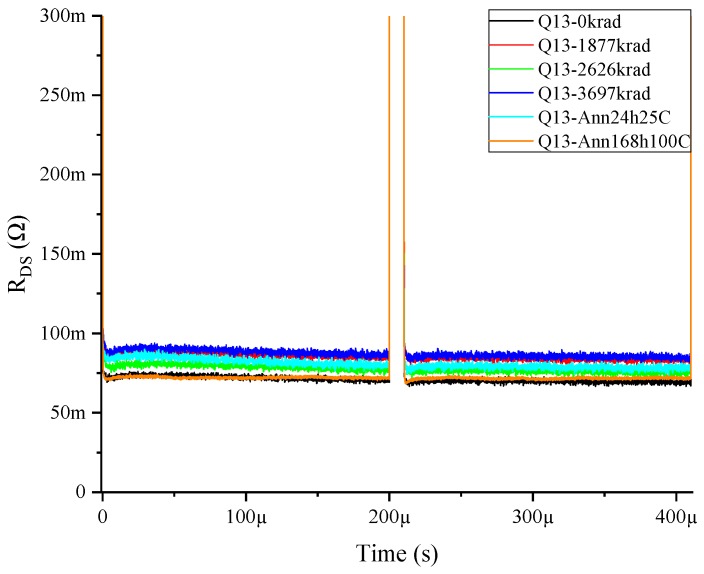
Dynamic resistance double-pulse measurements at 500 V for p-GaN Q13, subjected to drain and gate voltage during irradiation.

After the analysis of the results obtained by these double-pulse measurement tests, we found that for the HD-GIT HEMTs, there were no significant changes over the R_ON_dyn_ independent of the bias conditions applied during gamma irradiation. A proof of one measured sample is shown in Figure 6.

**Figure 7:**
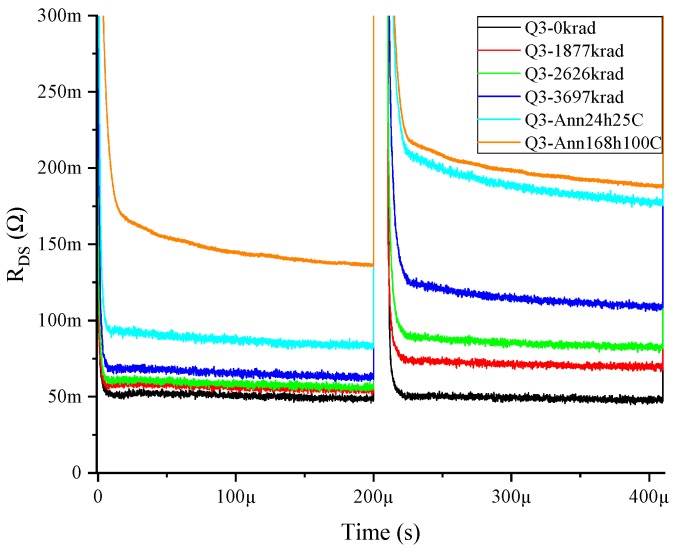
R_ON_dyn_ double-pulse measurements at 500 V for GaN MISHEMT Q3 subjected to drain and gate voltage during irradiation.

However, the GaN MISHEMTs suffered a change in its R_ON_dyn_ depending on the bias conditions applied during irradiation. An increase was evidenced when the MISHEMT was subjected to irradiation with drain and gate bias applied simultaneously. As an example of this behavior, the R_ON_dyn_ values of the device Q3 at different irradiation and annealing steps are shown in Figure 7. These devices suffered an increase in R_ON_dyn_ proportional to the accumulated dose. The total accumulated dose had a degradation effect on the device, which slightly increased the R_DSON_, but after the annealing process, an even higher degradation was observed in the channel resistance.

**Figure 8:**
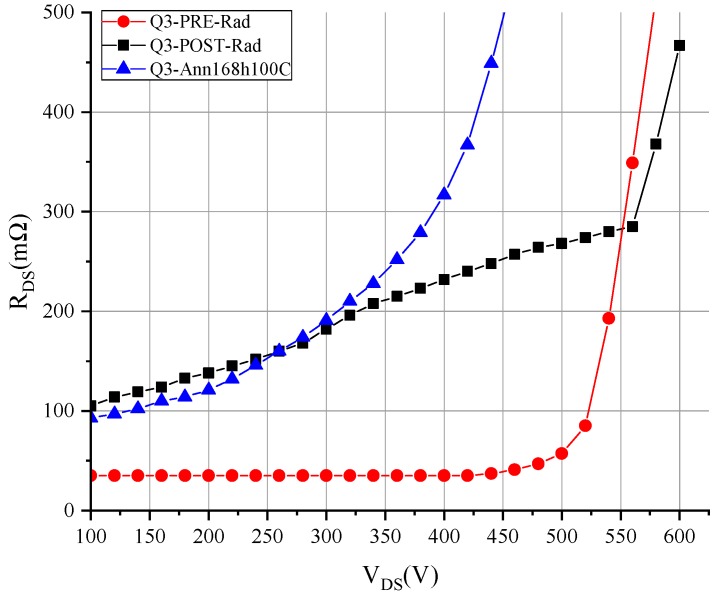
Multi-pulse test for GaN MISHEMT Q3 subjected to drain and gate voltage stress during irradiation.

The increase of R_ON_dyn_ was higher in the second pulses than in the first ones. This shows that trapping was induced by hot electrons during the semi-on-state in the switching events. Therefore, applying more switching pulses would increase the R_ON_dyn_. To demonstrate this behavior, the multi-pulse test was performed on this device. The result is shown in Figure 8, revealing the increase of the R_ON_dyn_ and providing information about different trapping phenomena. In the pre-irradiation measurement, the behavior was an exponential increase of the R_ON_dyn_ that started at a voltage stress of around 450 V. In the post-irradiation measurement, a new trapping behavior occurred, which had a linear dependence on the voltage applied from 100 to 550 V.

**Figure 9:**
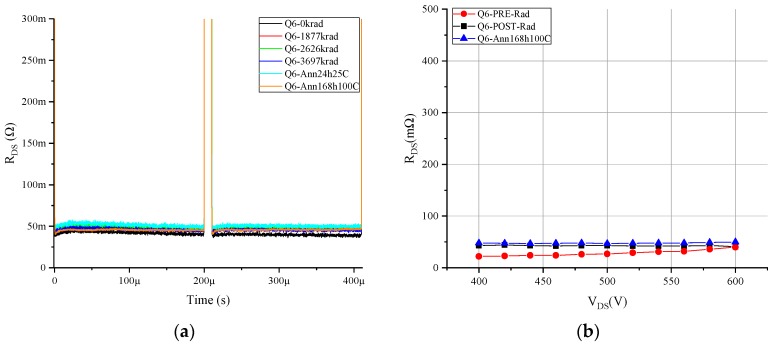
R_ON_dyn_ measurement for GaN MISHEMT Q6 subjected only to drain voltage during irradiation: (**a**) double-pulse measurement at 500 V, and (**b**) multi-pulse test measurement.

**Figure 10:**
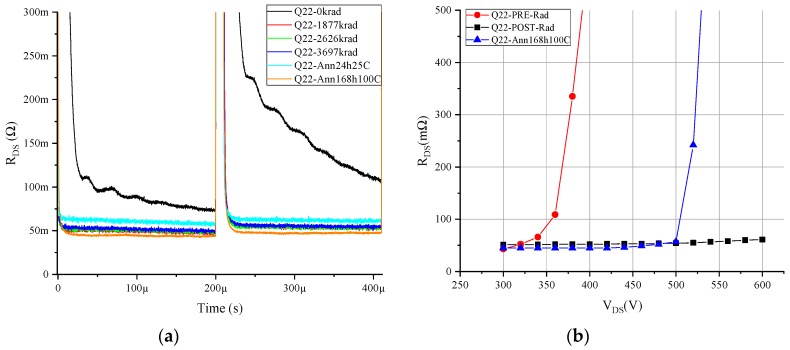
R_ON_dyn_ measurement for GaN MISHEMT Q22 subjected only to drain voltage during irradiation: (**a**) double-pulse measurement at 500 V, and (**b**) multi-pulse test measurement.

Furthermore, after the high-temperature annealing step, the R_ON_dyn_ exponential behavior was displaced to lower voltages, which further increased the R_ON_dyn_. On the other hand, the MISHEMT devices with shorted terminals or only drain voltage stress during irradiation did not suffer an increase in R_ON_dyn_, as shown in Figure 9 for device Q6. Moreover, the devices that had a high R_ON_dyn_ before irradiation suffered a decrease of the R_ON_dyn_ with the accumulated dose, as shown in Figure 10 for device Q22. 

From all the R_ON_dyn_ tests of the GaN MISHEMT, two conclusions can be drawn. First, the hole trapping induced by gamma radiation in the defects on the insulator compensated the initial electron traps existing in the devices. This is clearly shown in Figure 10a,b, where sample Q22 was irradiated with a 400 V bias condition, and as a consequence, the R_ON_dyn_ initially disappeared from the radiation exposure. This effect was observed in all samples subjected to irradiation with terminals shorted or only drain voltage applied.

The second conclusion is that the R_ON_dyn_ of the devices subjected to negative gate bias during irradiation increased. The multi-pulse test showed a linear increase of the R_DSON_ when stress voltage was applied. This effect resulted in different behavior than that from before irradiation, where the R_ON_dyn_ had an exponential dependency with the voltage applied before radiation. This effect, which is shown in Figure 8, matches with the assumption of electron trapping under the gate, which depletes the channel until the charges are removed by the gate current injection.

## 4. Discussion

The results confirmed the relevance of the GaN HEMT structure used, since the HD-GIT structure was robust in all of the steps of irradiation, with practically no changes in any of the properties measured. However, the MISHEMT structure suffered many changes. These differences were mainly due to two factors. The first is the removal of traps inside the HD-GIT device due to the use of an additional p-GaN region near the drain, which was electrically connected to the drain that effectively released the trapped charges. The second factor is the use of an insulator in the GaN MISHEMT. The insulator improved the gate performance, allowing a higher threshold voltage and reducing the gate leakage current, but in radiation environment conditions, the metal insulator has to be highly optimized; otherwise, some reliability problems can appear.

In this study, the results show that due to these differences, the GaN MISHEMT has different behavior depending on the bias applied during irradiation. In the case of shorted devices during irradiation, hole trapping in the insulator takes place, which means a reduction of the effectiveness of channel depletion. This hole trapping is due to the increase of the energy of electron–hole pairs due to radiation, which allows them to gain enough energy to become trapped in the gate dielectric [8,22]. This induces a negative threshold movement, an increase of the drain leakage current, an increase in gate current, and a reduction of the R_ON_dyn_ favored by the increase in the drain leakage current, as reported in [14].

The second bias condition studied was the devices subjected only to drain voltage. These devices experienced two phenomena: the damage on the insulator generating hole trapping, such as the shorted devices, which explains the same increase in the drain leakage current, gate current, and the decrease of R_ON_dyn_. However, the positive threshold movement of these devices was due to electron trapping at the surface, which is a common effect for these devices when submitted to high drain voltages.

When a negative gate voltage was also applied to the devices, both electron trapping on the surface due to the high drain voltage and electron trapping under the gate took place [23]. Therefore, on these devices, three effects took place together: the hole trapping in the damaged insulator, which induced an increase in gate current; the surface trapping due to the high drain voltage applied, which induced the positive threshold shift; and electron trapping under the gate due to the negative gate voltage applied, which induced a more negative shift of the gate current compared with the different biased devices. This trapping under the gate partially compensated the effects of the hole trapping in the insulator, resulting in these devices suffering less of an increase in drain leakage current. This also meant a reduction of the detrapping rate, which favored a greater increase in the R_ON_dyn_ instead of the decrease suffered by the different biased devices.

## 5. Conclusions

In this work, different behaviors were observed for GaN HEMTs subjected to gamma radiation which were structure-dependent. While HD-GIT HEMT characteristics were mainly unchanged during irradiation, the GaN MISHEMT structure underwent some changes. The results demonstrated that a degradation of the insulator took place during irradiation, which allowed hole trapping to induce a negative threshold voltage shift, an increase in the forward gate current and drain leakage current, and a reduction of the R_ON_dyn_. Additionally, the devices subjected to drain voltage during irradiation also suffered electron trapping on the surface due to the reduced barrier heights of the traps, which was the result of radiation inducing a positive threshold shift. In the case of devices biased with positive drain and negative gate voltages, they also suffered from trapping under the gate, which compensated the hole trapping in the insulator and forced an increase in the R_ON_dyn_. 

Therefore, the structure is one of the main factors that determines the reliability of GaN HEMTs under radiation, and here, the HD-GIT proved to be much more robust than the MISHEMT. This can be due to two factors. The first is the low reliability of the MISHEMT insulator during irradiation, as it is a weak region for the injection of traps. The existence of a p-doped region near the drain which removes the trapping is also crucial because the degradations reported were mainly due to trapping effects. In addition, different behaviors can take place in a MISHEMT when applying gamma radiation depending on bias condition. The results obtained here on the R_ON_dyn_ are necessary to consider when framing AlGaN/GaN radiation assurance tests, and they should be especially considered when using GaN HEMTs for power conversion units in future space missions.

## Figures and Tables

**Table 1:** Parameters of the investigated devices.

Parameter	Symbol	GaN MISHEMT GS66516T	p-GaN HEMT PGA26E07BA
**Drain-to-source breakdown voltage**	BV_DSS_	650 V	600 V
**Continuous drain current (Tc = 25 °C)**	I_D_	60 A	26 A
**Drain-to-source on-resistance (Tj = 25 °C)**	R_DSON_	25 mΩ	56 mΩ
**Input capacitance (1 MHz, 400 V)**	Ciss	520 pF	405 pF
**Total gate charge**	Q_G_	12.1 nC	5 nC

**Table 2:** Bias conditions applied during the irradiation test.

Condition	Device Type	Sample Serial Number	Units	Radiation	Gate Bias ^1^	Drain Bias ^1^
Control	MISHEMT	Q0	1	No	N/A	N/A
	p-GaN HEMT	Q25	1	No	N/A	N/A
Shorted	MISHEMT	Q11, Q12	2	Yes	0 V	0 V
	p-GaN HEMT	Q23, Q24	2	Yes	0 V	0 V
Drain bias	MISHEMT	Q6–Q10, Q21, Q22	7	Yes	0 V	400 V
	p-GaN HEMT	Q18–Q20	3	Yes	0 V	400 V
Drain–gate bias	MISHEMT	Q1–Q5	5	Yes	−5 V	400 V
	p-GaN HEMT	Q13–Q17	5	Yes	−5 V	400 V

^(1)^ Bias condition during the irradiation exposure (Not Applicable—N/A—to unirradiated control samples).

**Figure A1:**
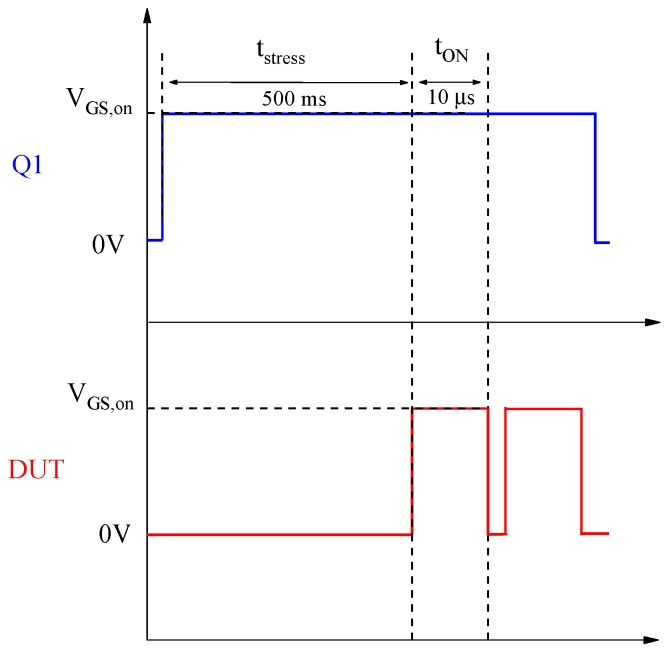
Gate voltage sequence for the double-pulse test.

**Figure A2:**
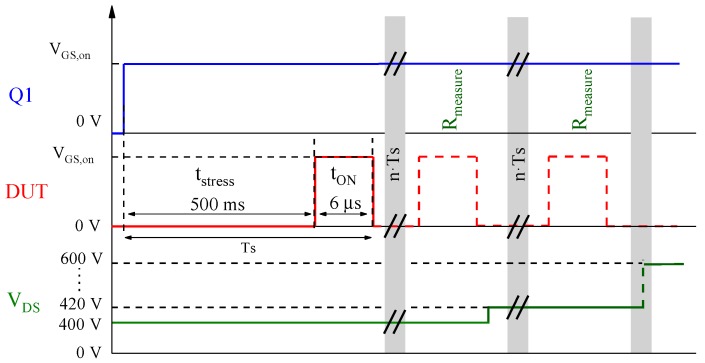
Gate voltage in both devices and drain voltage in the DUT for the multi-pulse test.

## Appendix A

The test sequence of the double-pulse measurement is shown in Figure A1. This sequence consists of stressing the device with a drain voltage for 60 s while it is in the off-state and then switching the device to the on-state in two pulses of 200 μs, separated by an off-state of 10 μs. These consecutive pulses following the voltage stress provide information about the main trappings, which are the trappings induced by voltage stress and the hot electron effect in the switching events [24]. For all tests, the drain voltage stress was 500 V, which represents a derating of 80% as it is fixed [25].

This test allows measuring the R_ON_dyn_ for a constant value of voltage stress. It has the advantage of allowing faster tests to be carried out on all the devices within the irradiation stops in less than 2 h (as is mandatory according to ESA rules [19]). However, it has the disadvantage of only providing information on the R_ON_dyn_ for 500 V off-stress. Therefore, when the time during the irradiation step test campaign is not critical (during the pre-irradiation measurements and the post-annealing), the multi-pulse test is also conducted because it provides information on R_ON_dyn_ behavior for a wide range of voltages stress. The sequence for this test is shown in Figure A2.

In this test, multiple pulses are applied with a stress time of 500 ms and an on-time of 6 µs for a given initial drain-to-source voltage applied to the DUT during off-time. The multi-pulse sequence finishes once the stationary value of the R_DSON_ is achieved. Afterwards, the test is repeated for a higher V_DS_ voltage, providing a new point of the R_DSON_ versus V_DS_ plot.
